# RIG-I overexpression decreases mortality of cigarette smoke exposed mice during influenza A virus infection

**DOI:** 10.1186/s12931-017-0649-z

**Published:** 2017-09-02

**Authors:** Xiaoqiu Wang, Wenxin Wu, Wei Zhang, J. Leland Booth, Elizabeth S. Duggan, Lili Tian, Sunil More, Yan D. Zhao, Ravindranauth N. Sawh, Lin Liu, Ming-Hui Zou, Jordan P. Metcalf

**Affiliations:** 1 Pulmonary and Critical Care Division, Department of Medicine, University of Oklahoma Health Sciences Center, Oklahoma City, OK USA; 2 The Lundberg-Kienlen Lung Biology and Toxicology Laboratory, Department of Physiological Sciences, Center for Veterinary Health Sciences, Oklahoma State University, Stillwater, OK USA; 3 Department of Biostatistics and Epidemiology, University of Oklahoma Health Sciences Center, Oklahoma City, OK USA; 4 Department of Pathology, University of Oklahoma Health Sciences Center, Oklahoma City, OK USA; 5 Veterans Affairs Medical Center, Oklahoma City, OK USA; 6 Center of Molecular and Translational Medicine, Georgia State University, Atlanta, GA USA; 7 Department of Microbiology and Immunology, University of Oklahoma Health Sciences Center, Oklahoma City, OK USA

**Keywords:** Influenza virus, Lung, Smoking, Cytokine, RIG-I, Transgenic mouse

## Abstract

**Background:**

Retinoic acid-inducible gene I (RIG-I) is an important regulator of virus-induced antiviral interferons (IFNs) and proinflammatory cytokines which participate in clearing viral infections. Cigarette smoke (CS) exposure increases the frequency and severity of respiratory tract infections.

**Methods:**

We generated a RIG-I transgenic (TG) mouse strain that expresses the RIG-I gene product under the control of the human lung specific surfactant protein C promoter. We compared the mortality and host immune responses of RIG-I TG mice and their litter-matched wild type (WT) mice following challenge with influenza A virus (IAV).

**Results:**

RIG-I overexpression increased survival of IAV-infected mice. CS exposure increased mortality in WT mice infected with IAV. Remarkably, the effect of RIG-I overexpression on survival during IAV infection was enhanced in CS-exposed animals. CS-exposed IAV-infected WT mice had a suppressed innate response profile in the lung compared to sham-exposed IAV-infected WT mice in terms of the protein concentration, total cell count and inflammatory cell composition in the bronchoalveolar lavage fluid. RIG-I overexpression restored the innate immune response in CS-exposed mice to that seen in sham-exposed WT mice during IAV infection, and is likely responsible for enhanced survival in RIG-I TG mice as restoration preceded death of the animals.

**Conclusions:**

Our results demonstrate that RIG-I overexpression in mice is protective for CS enhanced susceptibility of smokers to influenza infection, and that CS mediated RIG-I suppression may be partially responsible for the increased morbidity and mortality of the mice exposed to IAV. Thus, optimizing the RIG-I response may be an important treatment strategy for CS-enhanced lung infections, particularly those due to IAV.

## Background

Influenza A virus (IAV), a negative-sense single strand RNA virus, is a highly contagious agent that causes upper and lower respiratory tract infection resulting in 200,000 hospitalizations and 36,000 deaths in the United States per year [1, 2]. IAV is responsible for seasonal epidemics and, infrequently, global pandemics. In 2009, a new pandemic caused by an H1N1 influenza strain emerged and spread globally, the first influenza pandemic in more than 40 years.

Although recognizing and responding to pathogens in a non-specific manner, the innate immune system provides immediate protection against infection. Cells of the innate immune system detect viral infection largely through pattern recognition receptors (PRRs) present either on the cell surface or within distinct intracellular compartments. The innate immune system responds to influenza through three classes of PRRs. The member of the first class of PRRs that most cells use to detect IAV is the cytosolic sensor, retinoic acid inducible gene I (RIG-I) [3]. The second PRR class, endosomal Toll-like receptors (TLRs), are also involved. TLR3, a double-strand RNA sensor, may be used by some epithelial cells to detect the viral replicative intermediate dsRNA [4]. Immune cells such as macrophages and dendritic cells (DCs) that are also present in the respiratory system, detect viruses using PRR’s, and produce proinflammatory cytokines upon activation. Myeloid DCs mainly sense IAV through RIG-I and TLR3, while plasmacytoid dendritic cells (pDCs) use TLR7 to recognize influenza genomic RNA upon release in late endosomes [5]. Finally, the third class of PRRs, the nucleotide-binding domain and leucine-rich-repeat-containing proteins (NLRP), including NLRP3, and nucleotide-binding oligomerization domain 2 (NOD2), may serve as intracellular mediators of IAV initiated host-cell signaling via the regulation of caspase-1 [6–8]. NLRP are mainly involved in regulation of IL-1β maturation through the formation of a biochemical complex called the inflammasome [9]. The inflammasome regulates activation of caspase-1, and the subsequent cleavage of the IL-1β and IL-18 precursors into their functional forms, which are then released from the cell [6].

PRRs are critical for recognition of IAV and initiation of the early antiviral response through induction of interferons (IFNs) and proinflammatory cytokines that contain the infection [10]. Activation of these PRR receptors is the primary signal for production of proinflammatory cytokines and chemokines which are released to recruit and activate leukocytes [11]. To establish a productive infection and cause disease, IAV must overcome host innate immune responses that are rapidly activated during infections. The acute surge of cytokine release leads to intense infiltration with, and activation of, inflammatory cells. Highly pathogenic IAV strains, including pandemic strains and avian influenza, are usually associated with excessive cytokine responses [12, 13].

RIG-I is essential for IFN induction during RNA virus infections of non-pDC cell-types, and mice that are deficient in RIG-I-like receptor signaling pathways are extremely susceptible to RNA viruses [14–16]. Cigarette smoke (CS), as well as second-hand smoke, increases the susceptibility to pulmonary infection with pathogens including IAV, rhinovirus, respiratory syncytial virus and various bacterial pathogens [17–19]. In particular, epidemiological studies have shown that influenza infection is seven times more frequent and is much more severe in smokers than nonsmokers [20]. We have shown that CS suppresses the RIG-I gene and protein expression in human and mouse lung, and inhibits the antiviral innate immune response [21–23]. In vitro studies demonstrated that overexpression of RIG-I in guinea pig cell lines inhibited avian IAV H5N1 replication [24]. In this report, we generated a RIG-I transgenic (TG) mouse strain that expresses the RIG-I gene product under the control of the lung-specific surfactant protein C (SPC) promoter to determine if RIG-I overexpression improves the host innate response and outcomes during IAV infection in vivo.

## Methods

### Preparation of influenza virus stock and plaque assays

H1N1 influenza virus, A/PR/34/8 (PR8), was passaged in Madin–Darby canine kidney (MDCK) cells. Virus was grown in MDCK cells in DMEM/F12 with ITS+ (BD Biosciences, Franklin Lakes, NJ) and trypsin, harvested at 72 h postinfection, and titered by plaque assay in MDCK cells. There was no detectable endotoxin in the final viral preparations used in the experiments as determined by limulus amebocyte lysate assay (Cambrex, Walkersville, MD). The lower limit of detection of this assay is 0.1 EU/ml or approximately 20 pg/ml LPS. For determination of viral titers in infected mice, whole mouse lungs were collected and homogenized in 1 ml of ice cold PBS. Solid debris was pelleted by centrifugation and viral titer was determined using a standard plaque assay on MDCK cells [22]. Results were expressed as PFU/ml of extract.

### Animals

The pSPCA plasmid was a generous gift from Dr. G. Hoyle (University of Louisville). It contains a 3.7 kb fragment from the human SPC promoter and a 0.5 kb fragment containing the SV40 intron and polyA site. We used this plasmid to create an SPC-RIG-I DNA construct containing promoter sequences from the human SPC gene, and coding sequences from the mouse RIG-I cDNA. The mouse RIG-I cDNA was excised from the pBabe-puro-RIG-I vector, a gift from Dr. Maya Shmulevitz (University of Alberta), with Sal I and Cla I and cloned downstream of the SPC promoter. Transgenic mice were generated by microinjection of the linear SPC-RIG-I construct into fertilized C57BL/6 one-cell mouse embryos.

All mice were genotyped and bred under pathogen-free conditions in the animal facility at the Oklahoma University Health Sciences Center. Mice were housed at 20 °C on a 12 h light/dark cycle in sterile microisolator cages and fed ad libitum with sterile chow and water. The Institutional Animal Care and Use Committee of the Oklahoma University Health Sciences Center approved all of the protocols for the animal experiments.

### Whole-body CS exposure

Whole-body CS exposure was performed as described [22]. Mice were exposed to the smoke of 3R4F reference cigarettes (University of Kentucky, Lexington, KY) for 5 h per day. Mice receiving CS were gradually brought up to the target exposure over a period of 2 weeks, and treated 5 days/week for 6 weeks. Treatment was administered by placing mice in a Plexiglas smoking chamber (Teague Enterprises, Davis, CA). Smoke exposure was standardized to total suspended particles = 90 mg/m3, carbon monoxide = 350 ppm, 11% mainstream and 89% sidestream smoke in the chamber of the machine. These parameters were verified by determining carboxyhemoglobin (COHb) levels using an IL-682 CO-Oximeter (Instrumentation Laboratories, Lexington, MA). “Nonsmoking” (NS) treatment groups were conducted for the same periods of time, but mice were exposed to filtered room air.

### Influenza virus infection

IAV infection was performed under isofluorane anesthesia. IAV PR8 stock was diluted in PBS to make lethal and non-lethal doses of the virus. The mice were infected with IAV immediately after the last CS exposure. The virus solutions (50 μl) were administered by intranasal instillation as the animal was held in a vertical position. Control animals received PBS. For the survival test, the mice were monitored daily for 16 days, and clinical symptoms (shaking, lethargy, piloerection) and weight recorded daily.

### Bronchoalveolar lavage (BAL)

Mice were sacrificed using isofluorane. BAL was performed using a closed thorax technique by exposing the trachea, nicking the bottom of the larynx and inserting a 3/4-in. 22-gauge cannula into the proximal trachea. The proximal end of the trachea was tied off, and 0.6 ml of sterile PBS was gently introduced into the lungs and recovered. This was repeated 3 times for a total instilled volume of 1.8 ml. Return volume varied by <10% between samples. BAL fluid (BALF) was centrifuged to remove cells. Cells obtained were plated on slides using a Cytopro Cytocentrifuge (Wescor, Logan, UT) and stained with DiffQuik (Dade Behring, Newark, DE) for determination of cell populations. Differential counts were made with ≥400 cells/sample using 2 slides/mouse. The BALF was pooled and frozen.

### Multiplex immunoassay

Cytokine protein levels in the BALF and serum were determined by multiplex immunoassay (Affymetrix, Santa Clara, CA). The assay was run on a Bio-Plex 200 multiplex system (Bio-Rad, Hercules, CA).

### RIG-I protein levels by immunoblotting

The mouse lungs were harvested and homogenized, and then lysed in 500 μl of cold lysis buffer (150 mM NaCl, 50 mM Tris, pH 8.0, 10 mM EDTA, NaF, sodium pyrophosphate, 1% NP-40, 0.5% sodium deoxycholate, 0.1% SDS, and 10 μg of leupeptin/ml). Lung homogenates were clarified by centrifugation at 10,000×g, at 4 °C for 10 min, and the clarified lysates were mixed with SDS-PAGE sample buffer (60 mM Tris, pH 6.8, 10% glycerol, and 2.3% SDS) and heated to 95 °C for 5 min. The samples were separated by electrophoresis using a 4–15% gradient gel and transferred to polyvinylidene fluoride (PVDF) membranes. For the detection of proteins, the membranes were immunoblotted with rabbit polyclonal antibody specific for RIG-I (Abcam, Cambridge, MA) and glyceraldehyde 3-phosphate dehydrogenase (GAPDH; R&D Systems). The membranes were incubated with horseradish peroxidase-labeled goat anti-rabbit IgG (Cell Signaling Technology, Beverly, MA) and chemiluminescent reagents (Pierce Biotechnology, Rockford, IL). Blots were developed using the Syngene G:box Bioimaging System and GeneTools software (Syngene, Frederick, MD) and quantified using ImageQuant software (BD/Molecular Dynamics, Bedford, MA).

### Measurement of mRNA expression by quantitative real-time PCR (qRT-PCR)

Total RNA from lung was extracted using a modified TRIzol (Invitrogen, Carlsbad, CA) protocol and spectrophometrically quantitated. The integrity of RNA was verified by formaldehyde agarose gel electrophoresis. Equal amounts (1 μg) of RNA from each sample were reverse-transcribed into cDNA with the oligo (dT) SuperScript II First-Strand Synthesis System for RT-PCR (Invitrogen, Carlsbad, CA). Gene specific primers for mouse PRRs, cytokines and the β-actin housekeeping genes were used. The primers’ sequences are shown in Table 1. qRT-PCR was performed using 100 ng sample RNA and SYBR Green (Quanta Biosciences, Gaithersburg, MD) in a Bio-Rad CFX96™ Touch Real-Time PCR Detection System. Results were calculated and graphed using the ΔCT of the target gene and normalizer, β-actin.

**Table 1:** List of primers used in RT-PCR

Gene	Forward primer (5′-3′)	Reverse primer (5′-3′)
RIG-I	ATTGTCGGCGTCCACAAAG	GTGCATCGTTGTATTTCCGCA
IFN-β	CCATCAACTATAAGCAGCTCCAGC	CCACCATCCAGGCGTAGCTGTTG
β-actin	CAGAAGGACTCCTATGTGGGTG	GGATCTTCATGAGGTAGTCTGTC
TLR3	CTGGAGCCAGAACTGTGCC	GTTCTTGGAGGTTCTCCAG
IL-6	CCGGAGAGGAGACTTCACAG	GGTACTCCAGAAGACCAGAGG
IFN-λ 2/3	AGCTGCAGGCCTTCAAAAAG	TGGGAGTGAATGTGGCTCAG
TLR7	TTGGTTTGGGGTTTTTTTTG	ATTCCTGATAATGTCTTCTGGACA
NOD2	GGAAGGCACCCCATTGGGTTG	GCACAGCATGAACTTGGAGTCG
TNFα	GCCCAAGGCGCCACATCTCC	CCACTTGGTGGTTTGCTACG
IP-10	GGTCCGCTGCAACTGCATCC	GCAATTAGGACTAGCCATCC

### Histologic and immunohistochemical analysis of mouse lung tissue and type II alveolar epithelial cells (AEC II)

At day 6 after IAV infection, mice were sacrificed and lungs were fixed in 4% paraformaldehyde in PBS at room temperature for 30 min, and were then embedded in paraffin. Fixed tissue was hematoxylin and eosin (H & E) stained to assess inflammation and fibrosis. Sections (3–5 μm) were mounted on glass slides and immunoprobed with a rabbit anti-mouse polyclonal antibody for RIG-I (Abcam) or an anti-nucleoprotein (NP) polyclonal antibody [25].

AEC II were purified and plated on collagen coated glass slides and cultured [26]. After 1 day, the cells were infected with IAV PR8 at an MOI of 6 and incubated for an additional 24 h to stimulate RIG-I production. The cells were probed with a rabbit anti-mouse polyclonal antibody for RIG-I and a goat anti-mouse polyclonal antibody from SPC (Santa Cruz Biotechnology). Nuclei were stained with DAPI (blue). After washing, the sections were probed with a donkey anti-rabbit secondary antibody conjugated to Alexa Fluor 546 or a donkey anti-goat Alexa-Fluor 488 (all from BD/Molecular Probes). Transmitted light and fluorescent microscopy images were obtained using an Olympus BX51 microscope running Cellsens imaging software (Olympus, Center Valley, PA).

### Statistical analysis

Where applicable, the data was expressed as the mean ± standard error of the mean (SEM). Statistical significance was determined by one-way ANOVA with Student-Newman-Keuls post hoc correction for multiple comparisons. Survival significance was determined by Kaplan-Meier estimator analysis. Significance was considered as *p* < 0.05.

## Results

### Generation of RIG-I transgenic (TG) mice

**Fig. 1:**
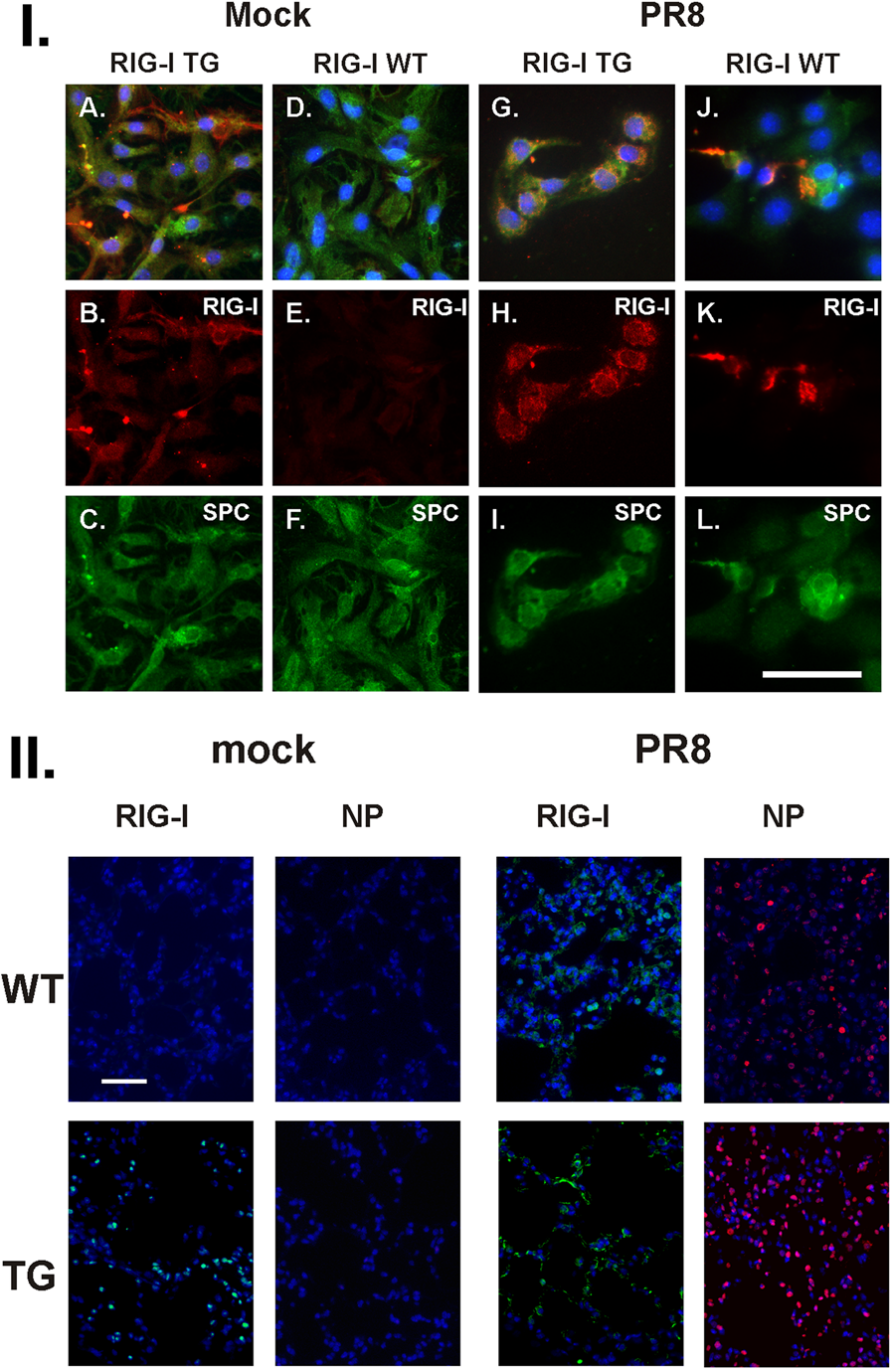
RIG-I and IAV NP expression in RIG-I TG and WT mice. **I** IAV-infected RIG-I TG and WT mice express RIG-I in isolated type II alveolar epithelial cells (AEC II). AEC II were infected with IAV PR8 at an MOI of 6 and incubated for an additional 24 h to stimulate RIG-I production. The cells were processed for immunohistochemistry (A-L) for detection of RIG-I (red, B, E, H and K) and surfactant protein C (SPC, green, C, F, I and L). Nuclei were stained with DAPI (blue) and were shown in overlaid images with RIG-I and SPC (A, D, J and I). Scale bars = 50 μm. **II** Mice were first intranasally infected with IAV PR8 (300 pfu/mouse) or mock infected with PBS. Mouse lungs were processed for immunohistochemistry for detection of RIG-I protein (green) or IAV NP (red). The lungs of 3 mice from each treatment group were processed for immunohistochemistry, and results shown were typical for the groups. The bar represents 100 μm for all the images

All mouse strains used were genotyped and bred under pathogen-free conditions in the animal facility at the Oklahoma University Health Sciences Center. We generated a C57BL/6 transgenic mouse strain that overexpressed the RIG-I gene product under the control of the lung-specific SPC promoter. The SPC promoter directs expression of transgenes in the distal lung epithelium, including type II alveolar epithelial cells (AEC II) and Club cells of the terminal bronchioles [27]. Thus, overexpression of RIG-I should be confined to these cells. To confirm expression in the alveoli, we isolated AEC II from RIG-I TG and wild-type (WT) mice. Cells were cultured for 2 days, infected with or without IAV, and stained with T1α (AEC I marker), SPC (AEC II marker), RIG-I, and DAPI (Fig. 1I). The results showed that at day 2, all of the cultures stained positive for SPC (Fig. 1I C, F, I and L). IAV-infected or uninfected RIG-I TG and IAV-infected WT AEC expressed high levels of RIG-I, while mock infected WT AEC did not express this protein (Fig. 1I, B, E, H and K). The data demonstrate that RIG-I protein is overexpressed in SPC rich AEC II in the lungs of TG mice that we generated.

We then confirmed RIG-I expression in mouse lung in vivo by immunostaining. Mice were infected with IAV and sacrificed after 6 days. PBS was used as a negative (mock) control for infection. Lungs were processed for immunohistochemistry for detection of IAV nucleoprotein (NP) and RIG-I. The PBS mock control had minimal immunofluorescence when stained for RIG-I in WT mice (Fig. 1II, left panels), while RIG-I was expressed in TG mice without IAV infection, as expected. RIG-I was highly induced in IAV-infected cells in WT and TG mice. Viral NP expression was present in mice infected with IAV (Fig. 1II, right panels). There was more staining for NP in TG mice than in WT mice. The results demonstrate that virus replication occurred in all mouse lungs after IAV infection with concurrent induction of RIG-I.

### RIG-I overexpression improved survival and reduced weight loss from IAV infection in CS-exposed mice

**Fig. 2:**
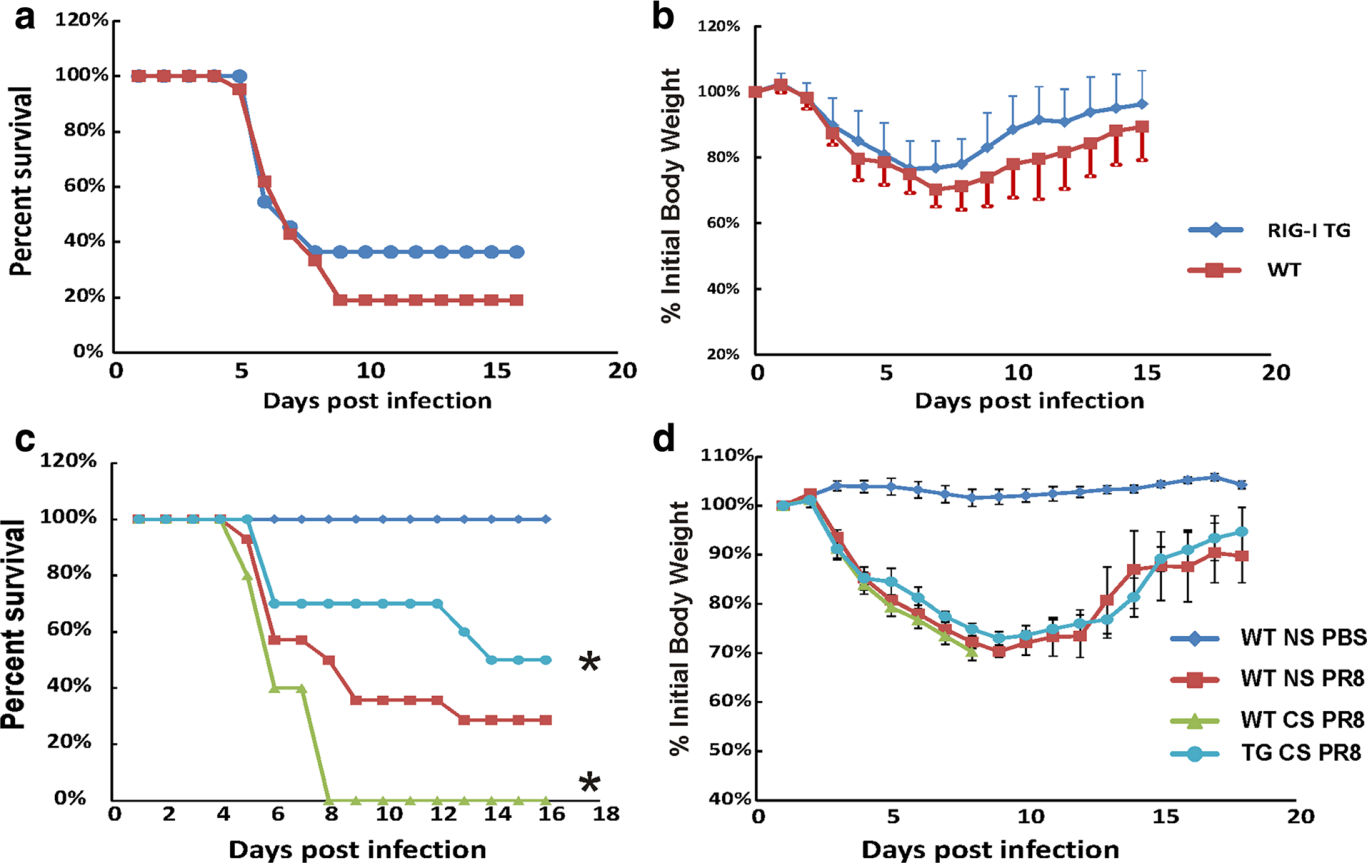
RIG-I overexpression improved survival and reduced weight loss due to CS exposure in IAV infection. **a** and **b** WT and littermate RIG-I TG mice were intranasally inoculated with a lethal dose of IAV (2000 pfu/mouse) without CS exposure. **c** and **d** WT and littermate RIG-I TG mice were exposed to CS or not for 6 weeks in a smoke exposure chamber (Teague Enterprises), then CS-exposed mice and NS WT mice were intranasally inoculated with a lethal dose of IAV (1000 pfu/mouse). Mortality (**a** and **c**) and body weights (**b** and **d**) were monitored daily. Body weight data were normalized to each mouse’s starting body weight. Data are expressed as mean ± standard deviation. * denotes a significant difference between the two groups by Kaplan-Meier survival test (*P* < 0.05)

In order to determine the in vivo role of RIG-I in severe IAV infection, we inoculated RIG-I TG and littermate WT animals with a lethal dose (2000 pfu/mouse) of IAV PR8. This dose was selected to cause approximately 80% mortality in WT mice. Overexpression of RIG-I in the lung reduced mortality during high dose viral infection (Fig. 2a). Specifically, RIG-I TG mice had an enhanced survival (approximately 36%, *n* = 11) compared to WT animals (19%, *n* = 17). Weight loss was significant in all infected groups and reached a nadir at 7 days after infection (Fig. 2b). The body weight data correlated with the survival data in that the lower survival groups lost more weight than groups with increased survival (approximately 23% vs. 31% loss for TG vs. littermate WT).

CS exposure increased morbidity and mortality of IAV infection in mice [28]. We sought to compare the effect of CS on mortality and weight loss due to IAV infection in the RIG-I TG and WT mice. Whole-body CS exposure was performed, as described [22]. Briefly, mice were exposed to the smoke of 3R4F reference cigarettes (University of Kentucky, Lexington, KY) for 5 h per day for 6 weeks. Exposure was accomplished using a smoking chamber (Teague Enterprises, Davis, CA). All mice were inoculated with a lethal dose (1000 pfu/mouse) of PR8 virus with PBS sham treated animals (mock) acting as a negative control. As shown in Fig. 2c, during IAV infection, CS-exposed WT mice had a lower survival rate than nonsmoking (NS) WT mice (approximately 0% for CS vs. 31% for NS mice). However, with the Kaplan-Meier survival analysis, CS-exposed RIG-I TG mice had a significantly improved survival rate compared to CS-exposed WT mice during IAV infection (55% for TG vs. 0% for WT mice, *p* < 0.05). CS-exposed RIG-I TG mice appeared to have enhanced survival compared to NS WT animals though this was not statistically significant (approximately 55% vs. 31%, *p* = 0.82). Morbidity also appeared to be improved by RIG-I overexpression, as IAV infected CS-exposed RIG-I TG mice had slightly less weight loss compared to IAV infected CS-exposed WT mice, though this did not reach statistical significance (Fig. 2d). Thus, RIG-I overexpression likely reduced weight loss and significantly improved survival in CS-exposed mice during IAV infection.

### RIG-I overexpression restored the CS suppressed inflammatory response profile in the lung during IAV infection

To investigate the role of RIG-I in the inflammatory response to IAV, RIG-I TG and WT mice with or without CS exposure were inoculated intranasally with a single, non-lethal dose of the IAV PR8 strain. To prevent the death of infected mice before sacrifice, a lower dose (300 pfu/mouse) was used. The mock group was sham inoculated with an equal volume of PBS as a negative control. Animals were sacrificed at 2, 4 and 6 days after infection, and bronchoalveolar lavage fluids (BALF) were collected to assess cellular infiltration and mediator content in the airspaces.

**Fig. 3:**
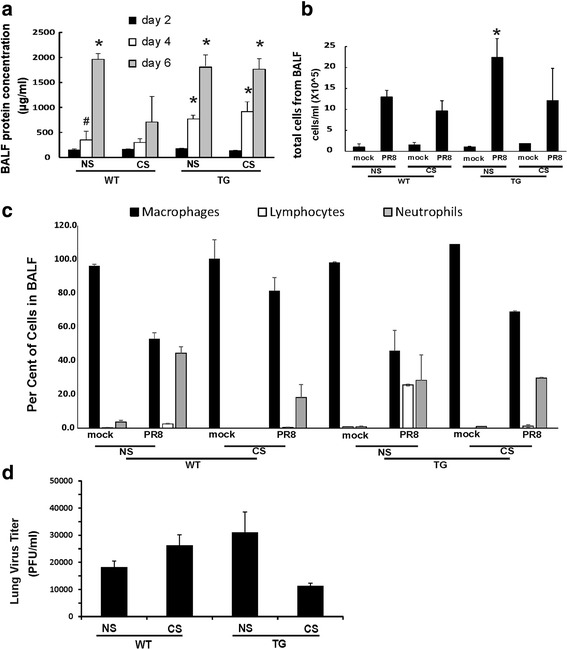
Inflammatory profile in the BALF and virus titer in the lung. For CS groups, RIG-I WT and TG mice were exposed to CS for 6 weeks in a smoke exposure chamber. Then the mice were intranasally infected with a non-lethal dose of IAV PR8 (300 pfu/mouse) or mock infected with PBS. BALF was harvested at the indicated time points after infection. Total protein levels (**a**), total cells (**b**) and immune cell differential (**c**) in BALF were determined. Lung tissue viral titers were determined at 6 days post-infection by plaque assay on MDCK cells (**d**). Data are expressed as means ± SEM (*n* = 9 per group). * denotes a significant difference compared to the PR8 infected WT CS group at corresponding time point (*P* < 0.05). # denotes no significant difference compared to the PR8 infected WT group

The inflammatory response was characterized first by examining total protein in BALF. The amount of total protein in BALF is an index of transudation from the vascular compartment into the lungs, and is also an indirect measure of the lung inflammatory response [29, 30]. CS treatment in WT mice significantly reduced BALF protein content at day 6 after infection (WT CS vs. WT NS, *p* < 0.05). In contrast, BALF protein levels in CS-exposed RIG-I TG mice were similar to those seen in NS mice after infection, and significantly higher than those seen in CS- exposed WT animals (Fig. 3a). Also, elevation in BALF protein levels occurred earlier in RIG-I TG mice than in WT mice. Starting at day 4, protein levels of both CS and NS RIG-I TG mice were significantly higher than WT mice (*p* < 0.05).

In terms of total inflammatory cells, IAV infection significantly increased the percentage of neutrophils in BALF in all infected groups (Fig. 3b, c). CS exposure appeared to decrease the total BALF cell number and the percentage of neutrophils during infection in WT animals. However, CS exposure of RIG-I TG mice did not change the neutrophil percentage in BALF during infection, and in the absence of CS, total cell numbers in RIG-I TG mice were the highest of any treatment group. These results indicated that higher mortality of WT CS mice was not mediated through uncontrolled inflammatory cell recruitment to the lung, but instead was more likely related to an impairment of recruitment mediated by CS. We note that we did not see significant numbers of eosinophils in any of the groups, though small numbers of these cells induced during infection may have been missed [31, 32]. Plaque assays of whole lung showed that CS exposure increased viral titers in WT animals at 6 days of infection, but this did not occur in RIG-I TG mice (Fig. 3d), though these differences were not statistically significant. Thus, although there was no direct correlation of mortality with lung viral burden, CS effects could be modulated through impairment of viral clearance that is reversed by RIG-TG overexpression.

**Fig. 4:**
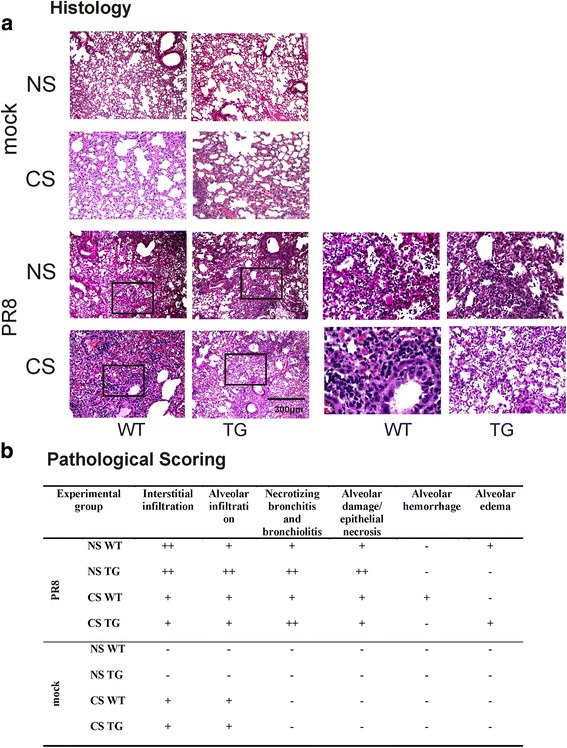
Mouse lung tissue pathology after IAV infection. Mice were intranasally infected with a non-lethal dose (300 pfu/mouse) of the IAV PR8 strain. Samples were harvested after 6 days. Lung tissue sections prepared from the infected mice were fixed, processed and stained with H&E (**a**). The lungs of three mice from each treatment group were processed for histology, and results shown were typical for the group. The right two columns were higher magnification images from the left corresponding frames. **b** Pathologic findings of inflammation and injury were graded for each treatment group

Examination of histopathology revealed that IAV infected lungs in all mice showed typical viral pneumonia with interstitial edema and inflammatory infiltration, varying degrees of acute intra-alveolar edema and/or hemorrhage, and necrotizing bronchitis and bronchiolitis. IAV infection resulted in the expected neutrophilic alveolar infiltrate with some lymphocytes (Fig. 4a). Histopathologic scoring of the cardinal features of IAV infection was evaluated by a pathologist blinded to the treatment groups from which the slides were obtained (R.N.S.). At day 6, PR8 infected NS RIG-I TG mice seemed to have slightly more overall alveolar damage and epithelial cell necrosis than the other groups (Fig. 4b). At day 4, PR8 infected CS-exposed WT mice had slightly more necrotizing bronchitis and bronchiolitis than the other groups (not shown). Otherwise, we found little difference in terms of the severity of inflammation in RIG-I TG and WT mice.

### CS-exposed RIG-I TG mice demonstrated an unimpaired innate response to influenza infection

**Fig. 5:**
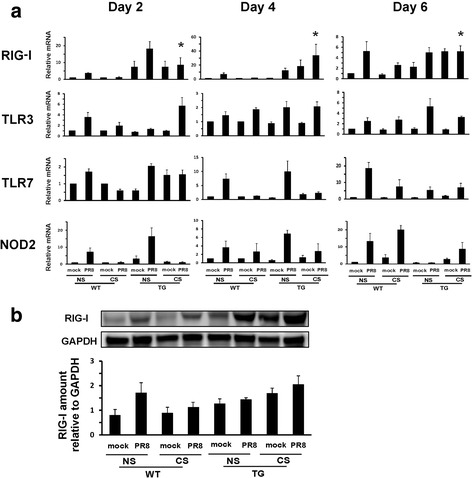
RIG-I mRNA and protein expression were restored during IAV infection in CS-exposed RIG-I TG mice. For CS groups, RIG-I WT and TG mice were exposed to CS for 6 weeks in a smoke exposure chamber. Mice were infected with a non-lethal dose (300 pfu/mouse) of the IAV PR8 strain. Mock treated mice were inoculated with PBS. At the indicated time points, the mice were sacrificed, and lung tissues were collected for RNA preparation and protein extraction. PRR mRNA (**a**) levels were assessed by qRT-PCR and normalized to β-actin. Data are expressed as means ± SEM (*n* = 9). **b** RIG-I protein levels were determined at day 6 post-infection by western blot and normalized to GAPDH. Data is representative of three separate experiments. * denotes a significant difference compared to the WT CS PR8 group (*P* < 0.05)

We measured PRR and cytokine expression during infection in all mouse groups. As in all non-mortality experiments, CS-exposed and NS mice were intranasally infected with 300 pfu IAV PR8. Lung tissues and BALF were collected at 2, 4 and 6 days after infection, and PRR and cytokine expression were determined by qRT-PCR or multiplex immunoassay, respectively. All tested PRR mRNA were induced by virus infection in lungs from WT NS mice from day 2 after IAV infection (Fig. 5a). CS exposure markedly suppressed RIG-I and TLR7 induction by IAV in WT mice at all time points. There was constitutive expression of RIG-I mRNA in TG mice even without IAV infection. RIG-I mRNA expression was increased in RIG-I TG mice as compared with WT mice at all days after infection, even with CS exposure. The induction of RIG-I by IAV PR8 and overexpression of RIG-I in TG mice were also confirmed at the level of protein expression by immunoblotting at 6 days post-infection (Fig. 5b).

**Fig. 6:**
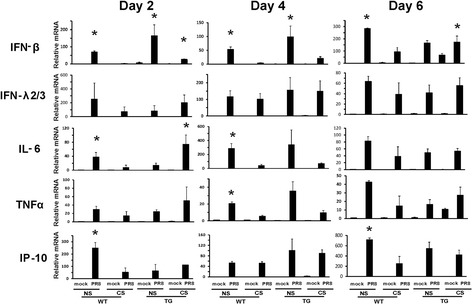
Antiviral and cytokine mRNA induction in the lung was restored in RIG-I TG mice during influenza infection. For CS groups, RIG-I WT and TG mice were exposed to CS for 6 weeks in a smoke exposure chamber. Mice were intranasally infected with a non-lethal dose (300 pfu/mouse) of the IAV PR8 strain. Mock treated mice were inoculated with PBS. At the indicated time points, the mice were sacrificed, and lung tissues were collected for RNA extraction. IFN and cytokine mRNA levels were assessed by qRT-PCR and normalized to β-actin. Data are expressed as means ± SEM (*n* = 9). * denotes a significant difference compared to the WT CS PR8 group (*P* < 0.05)

We also measured IFN and cytokine mRNA induction in response to IAV in these animals. Consistent with the protein content in BALF, mRNA expression of the proinflammatory cytokines IL-6, TNFα and IP-10 was highly induced among all groups during IAV infection (Fig. 6). However, at day 2 and day 4 after infection, the antiviral IFN-β and proinflammatory IL-6/TNFα response to flu infection was significantly inhibited in CS-exposed WT mice. At day 6, the induction of these three important cytokines was slightly increased by IAV infection but was still much lower in CS WT mice than in NS WT and TG mice. Specific effects of RIG-I overexpression included a significant increase in the antiviral IFN-β and proinflammatory IL-6 mRNA expression in IAV-infected CS TG mice over similarly treated WT mice (*p* < 0.05, both comparisons, Fig. 6). The data suggest that CS exposure inhibits the innate antiviral and proinflammatory immune response to IAV infection, while RIG-I overexpression reverses this immunosuppression.

**Fig. 7:**
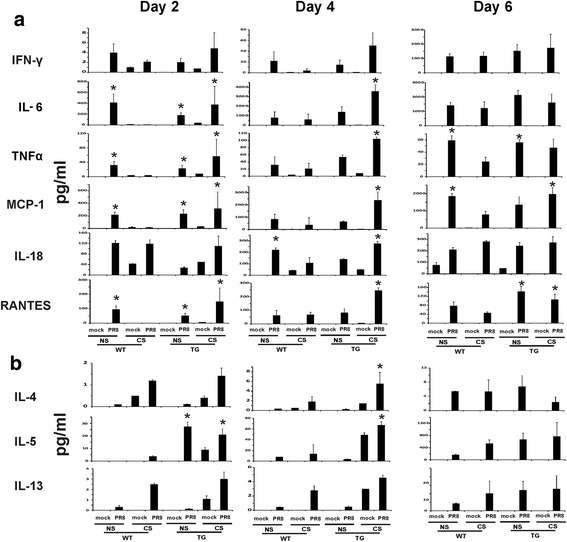
Antiviral and cytokine responses in BALF were restored in RIG-I TG mice during influenza infection. For CS groups, RIG-I WT and TG mice were exposed to CS for 6 weeks in a smoke exposure chamber. Mice were intranasally infected with a non-lethal dose (300 pfu/mouse) of the IAV PR8. BALF was harvested at the indicated time points after infection. Mock treated mice were inoculated with PBS. **a** Th1 and **b** Th2 cytokine protein levels in the BALF were determined by multiplex immunoassay. Data are expressed as mean ± SEM (*n* = 6 per group). * denotes a significant difference compared to the WT CS PR8 group (*P* < 0.05)

To confirm that cytokine induction was reflected at the level of translation, we measured cytokine proteins in BALF from mice exposed to IAV in all three genotypes using multiplex immunoassay (Fig. 7). At day 4, CS exposure reduced induction of IFN-γ, IL-6, MCP-1, TNFα and IL-18 protein in IAV infected WT mice. At day 6, induction of MCP-1, TNFα and RANTES was still suppressed in CS WT mice. Induction of IFN-γ, IL-6 and IL-18 may have been delayed by CS as day 6 levels were similar to those seen in WT NS mice. However, CS exposure did not impair or delay the IAV cytokine response in RIG-I TG mice, indicating that RIG-I overexpression supported a robust innate immune response to IAV. The antiviral cytokines were elevated in RIG-1 transgenic mice exposed to CS and infected with PR8 virus, and the early induction of those cytokines may play protective roles against mortality and morbidity.

## Discussion

The regulatory mechanisms of innate immunity are sophisticated and complex. RIG-I is an important PRR that senses viral infection and activates antiviral and proinflammatory cytokine defenses, which may limit viral replication and increases resistance to infection. Extensive evidence has demonstrated that RIG-I is a key sensor of infection by many RNA viruses. Recent work has also shown that RIG-I directly inhibits viral replication independent of antiviral signaling [33, 34]. Here, we compared the lung innate response in the lungs during IAV infection between RIG-I TG and WT mice. Our work showed that CS-exposed RIG-I TG mice had improved survival and lost less body weight compared to CS-exposed WT mice following IAV infection. CS-exposed IAV-infected RIG-I TG animals displayed a similar response level of lung inflammation as did NS WT mice. RIG-I overexpression restored the induction of antiviral and inflammatory genes, which were suppressed by CS in WT mice following intranasal challenge with IAV. This occurred despite the fact that inhibition of other PRRs by CS, for example TLR7, was not reversed by RIG-I overexpression (Fig. 5a). Thus, the host response initiated by RIG-I overexpression following IAV infection of mice could compensate for inhibition of multiple PRRs by long term smoking. The data suggest that RIG-I could play an important role in the recognition and control of IAV infection in mice, both in CS-exposed, and NS animals.

PRR induction by IAV in Fig. 5a was determined by mRNA level only. Additional PRR proteins or pathways likely play a role in IAV infection. Others have shown that the absence of either the RIG-I signaling adaptor mitochondrial antiviral-signaling protein (MAVS) [35] or TLR3 [36] diminishes viral clearance and adaptive immunity to IAV infection. These studies suggest that host recognition of IAV by PRRs in vivo and initiation of innate immunity is more complex than currently appreciated, and could differ from well-established mechanisms in vitro. There might be extensive cooperative and/or competitive interactions among different PRRs that support and regulate antiviral sensing and induction of innate immune responses.

During influenza infection, the severity of disease is the result of the interplay between viral virulence and the host response. Local inflammation and enhanced cytokine production are the hallmarks of host innate immune responses that act to protect against infection by viruses, and are essential in local control of invading microbes. The pneumonia that follows seasonal influenza infection is generally a rare complication and can be primary viral pneumonia, secondary bacterial infection, or a mixed viral/bacterial infection. By contrast, primary viral pneumonia is a major manifestation of human H5N1 disease [37]. Primary viral pneumonia was also prominent during the severe H1N1 pandemic of 1918. In these situations, aggressive and uncontrolled immune cytokine responses may lead to deleterious consequences [38]. For example, inflammatory responses in animal infection models immunologically naïve to IAV show that enhancement of inflammation in young adults may have been a major contributor to mortality during the 1918 influenza pandemic [39]. In mild infection, the host has a limited or moderate response with little tissue damage and, thus, the disrupted homeostasis is restored rapidly [40]. Either uncontrolled (usually in pandemic IAV infection) or suppressed (in immunosuppressed patients) inflammation could be dangerous to the host. Here, we demonstrate that CS suppressed and delayed the host response to IAV while RIG-I overexpression restored the impaired inflammatory and antiviral response in the lung. Thus, normal and appropriate inflammation may be necessary and beneficial for survival of the mouse during seasonal IAV infection. From Fig. 1.II, there was more staining for NP in TG mice than in WT mice, and we did not find a direct correlation of viral titer in the lung with mortality in these different groups (Fig. 3d), although it is possible this would be evident at other times during infection. Another possibility could be that the viral titers were measured during low dose IAV infections performed to study mechanisms, which may not completely mimic viral replication during fatal infection. Furthermore, enhanced viral burden may not be solely responsible for morality in mice [41].

Because innate immunity is a key determinant of the subsequent host immune response and clinical outcome, we evaluated the timeline of inflammation and cytokine expression in the lung during infection. Our results support the hypothesis that increased mortality in IAV infection is due to dysregulation of the antiviral response in CS mice. The study also suggested that critical events influencing outcomes may occur early after infection. Early gene activation elicited by IAV infection of RIG-I TG mice likely controls the lethality. We assume that the BALF protein levels seen in NS WT mice and in both sets of TG mice reflect a near optimal host inflammatory response, as they are associated with lower mortality. Therefore, the lower levels of BALF protein levels in CS-exposed WT mice reflects a deleterious modulation of the innate response.

Cytokines also have important effects on the adhesive properties of the endothelium, causing circulating leukocytes to stick to the endothelial cells of the blood vessel wall and migrate between them to the site of infection, to which they are attracted by chemokines. CS exposure impaired the host cytokine responses of IFN-γ, IL-6, MCP-1, TNFα, IL-18 and RANTES to IAV in the lung as compared with NS infected mice. In contrast, these cytokine responses were normalized in RIG-I TG mice exposed to CS. It is notable that RIG-I overexpression restored the immune response in a controlled fashion, as it did not increase deleterious inflammation. This may occur due to regulation of the RIG-I signal through sophisticated post-translational modifications resulting in a robust yet ‘tunable’ cytokine response to maintain immune homeostasis [42].

## Conclusion

This study is the first to describe the effect of RIG-I overexpression on innate responses to IAV in vivo. We demonstrate that RIG-I overexpression in the lung compensates for CS-mediated innate immunosuppression in the mouse and improves survival during viral infection. These results provide new insight into the mechanisms whereby RIG-I maintains homeostasis of the host immune system during influenza infections.

## Abbreviations

BAL: Bronchoalveolar lavage
BALF: Bronchoalveolar lavage fluid
CS: Cigarette smoke
DC: Dendritic cell
GAPDH: Glyceraldehyde 3-phosphate dehydrogenase
H & E: Hematoxylin and eosin
IAV: Influenza A virus
IFN: Interferon
MAVS: Mitochondrial antiviral-signaling protein
MDCK: Madin–Darby canine kidney
NLRP: Nucleotide-binding domain and leucine-rich-repeat-containing proteins
NOD2: Nucleotide-binding oligomerization domain 2
NP: Nucleoprotein
NS: Nonsmoking
PR8: A/PR/34/8
PRR: Pattern recognition receptor
RIG-I: Retinoic acid inducible gene I
SPC: Surfactant protein C
TG: Transgenic
TLR: Toll-like receptor
WT: Wild-type
